# The effectiveness of interventions to involve men living with HIV positive pregnant women in low-income countries: a systematic review of the literature

**DOI:** 10.1186/s12913-019-4689-6

**Published:** 2019-12-09

**Authors:** Isotta Triulzi, Ilaria Palla, Fausto Ciccacci, Stefano Orlando, Leonardo Palombi, Giuseppe Turchetti

**Affiliations:** 1 Institute of Management, Scuola Superiore Sant’Anna, Piazza Martiri della Libertà, 33, 56127 Pisa, Italy; 2 Department of Biomedicine and Prevention, University of Tor Vergata, Rome, Italy; 3 International University of Health and Medical Science, UniCamillus, Rome, Italy

**Keywords:** Male involvement, Attendance to prenatal care, Pregnant women, Antenatal clinic, Prevention of mother-to-child transmission, HIV, Low income countries, Health prevention

## Abstract

**Background:**

Male involvement (MI) along the continuum of HIV healthcare services has been promoted as a critical intervention in low-income countries and represents one of the reasons for dropout and low retention of women along the cascade of care. The present review aims to identify interventions adopted to improve MI across Antenatal Clinics (ANCs).

**Methods:**

For this systematic review, we searched electronic databases, including Scopus, PubMed, Web of Science (from 2008 to 2018) in English language. We included all interventions explicitly aimed at involving partners in pregnant women’s HIV continuum of care and we excluded studies performed in developed countries, not involving pregnant women. We followed the PRISMA checklist.

**Results:**

We identified a total of 1694 records and excluded 1651 after duplicates were removed and abstract eligibility assessments were performed. Forty-three full-text articles were screened, but only 12 studies were included. Recurrent intermediate outcomes were antenatal partner attendance rate and male HIV testing. We subdivided articles according to the type of intervention: single intervention (7) and multiple interventions (5). Among single interventions, two studies evaluated the use of an invitation letter sent via women to encourage male attendance to the ANC. Four Randomized Controlled Trials (RCTs) compared the invitation card (standard of care, SC) to word of mouth, information letter, home visit and invitation card plus partner tracing. The partner attendance rate was lower in SC than in the intervention arm in three RCTs: information letter (14.2% vs 16.2%), home-visit (39% vs 87%) and invitation card plus partner tracing (52% vs 74%). Home visit strategies seemed the most effective. One study evaluated words of encouragement adopted to trigger women to invite their partners.

Among multiple interventions, the most effective strategies in terms of male attendance included health promotion through education and healthcare worker development. These interventions were more likely to be effective in promoting MI than single interventions.

**Conclusions:**

From the review emerges the importance of male involvement in HIV cascade for pregnant women in countries with a significant HIV incidence and the need to define more precise indicators for measuring MI.

## Background

The term male involvement refers to “engaging men to participate in health services together with their partners, especially in Antenatal Clinical settings” [1]. Male involvement (MI) represents a critical issue in maternal and child healthcare services in developing countries [2] and is associated with improved maternal adherence and retention [3–5] and reduction of infant HIV infection [6–8].

The lack of male partner involvement along the continuum of HIV healthcare services represents one of the main reasons for treatment refusal, delayed enrolment, dropout and low retention of pregnant and breastfeeding women [2, 9, 10].

Although MI is a not well-defined concept and there is no a conventional way to evaluate and measure it, it is possible to identify gaps that affect men’s health-seeking behaviours and discourage from looking after themselves and their partners. The significant causes of non-involvement is due to cultural, societal and gender factors, socio-economic factors, health service barriers and policy gaps [5, 11, 12].

According to UNAIDS, despite social and economic advantages, men use to seek out health care less than women, both for HIV testing and for Antiretroviral Therapy (ART) therapy. In fact, across Sub-Saharan Africa (SSA), the knowledge of HIV status is lower in men and boys than in women and girls living with HIV [13].

The need to involve males has been increasingly recognized since 1990, but only in 2012 the World Health Organization set partner involvement as one of the priority interventions to improve PMTCT (Prevention of Mother-to-Child Transmission) outcomes [2]. Unfortunately, efforts to promote sexual health and health prevention campaigns are aimed exclusively at women [14–16]. In the scientific literature, many studies highlight the importance of including men in care to improve health outcomes of female partners, but very few interventions have been proposed and implemented on a large scale [17–19].

The present study aims to review and evaluate the effectiveness of the interventions adopted to improve male involvement in PMTCT as an intermediate outcome to improve effectiveness of HIV/AIDS treatment and prevention programs targeting women. Although the final outcome to which the interventions aim is women’s health, in this review we have considered as given the relationship between MI and women’s health and we have limited the analysis to the effectiveness of interventions in promoting MI.

## Methods

### Identification of the studies

We decided to perform a systematic review to evaluate the effectiveness of the existing interventions [20] using PubMed, Scopus and Web of Science. PubMed is an ideal tool that offers a quick free search with various keywords and it is readily update compared to Scopus and Web of Science. Given that PubMed is mainly focused on medicine and biomedical sciences, we searched though Scopus and Web of Science that cover social sciences and humanities and larger number of journals [21]. The electronic search strategy for PubMed was: ((HIV[Title/Abstract] OR AIDS[Title/Abstract]) AND (Male[Title/Abstract] OR men[Title/Abstract] OR man[Title/Abstract] OR husband[Title/Abstract] OR couple[Title/Abstract] OR partner[Title/Abstract])) AND (PMTCT[Title/Abstract] OR MTCT[Title/Abstract] OR mother-to-child transmission[Title/Abstract] OR vertical transmission[Title/Abstract] OR pPTCT[Title/Abstract] OR pregnant[Title/Abstract]) AND (“2008/10/01”[PDat]: “2018/09/28”[PDat] AND “humans”[MeSH Terms] AND English[lang]).

Furthermore, reference lists and key journals have been hand-searched. The search referred to the period October 2008–October 2018 adopting the search strategy indicated in the table (Additional File 1: Table S1).

We followed the fundamental principles of systematic review according to PRISMA 2009 (Preferred Reporting Items for Systematic Reviews and Meta-Analyses) checklist (Additional File 1: Table S2).

### Inclusion criteria

- Study Population: **HIV+ and HIV-** pregnant women and their male partners
- Interventions: all types of studies testing interventions and strategies to involve male partners in antenatal settings. We included all interventions explicitly aimed at involving partners in pregnant women’s HIV continuum of care.
- Outcomes: all the outcomes that measured male involvement
- Countries: low income countries around the world
- Language: English

### Exclusion criteria

We excluded studies not written in the English language and all papers published before 2008. Conference proceedings, case reports, systematic reviews, reviews, letters, and commentaries also were ruled out. We excluded studies where the component of MI was marginal.

### Study selection

We followed a three-step processes for the evaluation of the studies. First, one researcher screened titles and abstracts of all identified studies which have been double-checked by a second reviewer. Secondly, two reviewers applied independently inclusion criteria on the full texts of the obtained articles. A third researcher resolved discordance between reviewers. Finally, data were extrapolated by two reviewers and then cross-checked. Finally, extrapolated.

### Data items

The data extracted included the following items: Authorship, year of study, and journal; Country where the study was conducted; Objectives; Study design; Study duration; Sample size; Characteristics of participants; Results; Conclusion.

The meta-analysis was not feasible due to the heterogeneity of the studies (design, measured outcomes),

so we completed a narrative synthesis for each intervention.

We considered separately the included studies with multiple integrated components and studies specifically designed to assess the effect of a standalone male involvement intervention.

**Fig. 1:**
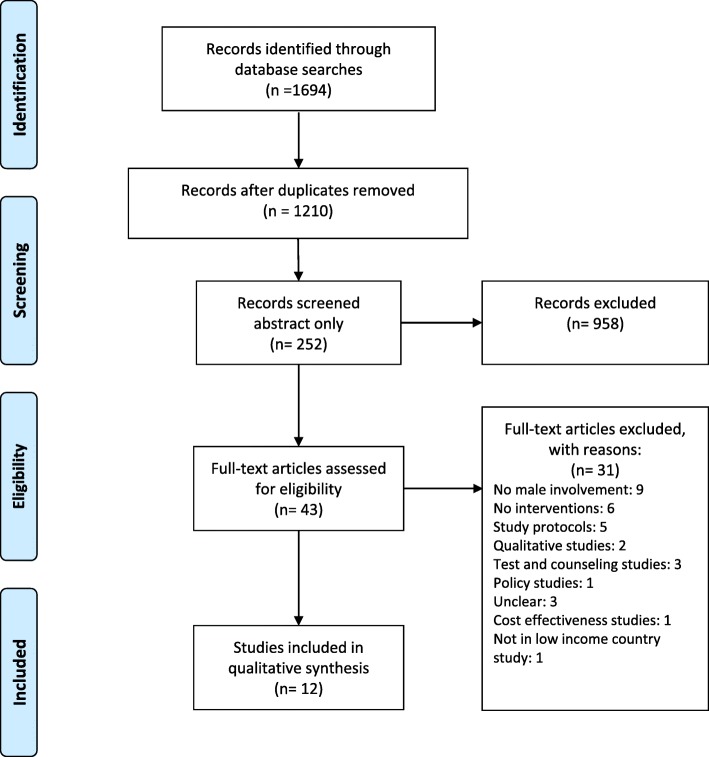
PRISMA Flow Diagram: research and selection of studies

The outcomes measures showed different dimensions depending on the design of the study: the rate of male attendance, Odd Ratio (OR), Risk Relative (RR) and Risk Difference (RD) (Fig. 1).

**Fig. 2:**
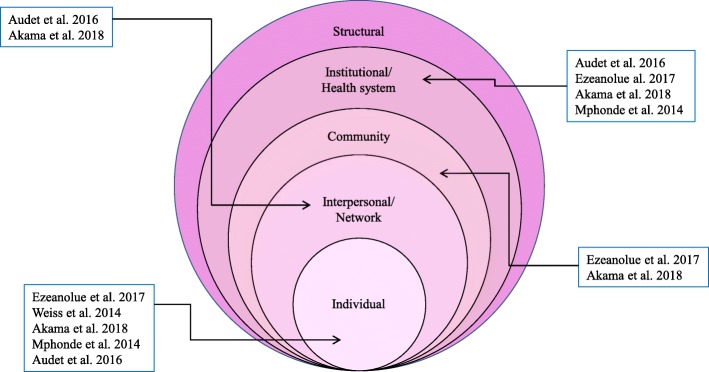
Effect of multiple integrated component interventions on Socio-Social ecological model adapted by Kaufman et al. 2004

## Results

### Study selection

We identified a total of 1694 papers from three databases. After removing the duplicates, we screened 1210 titles from which 252 abstracts met the inclusion criteria. The reasons for the exclusion were: the studies did not assess an intervention, they were qualitative studies, the study population was from high-income countries, they were focused on special population groups, and they were based on clinical trial not yet concluded.

Therefore 43 full-text articles were initially included in the review. Of these 31 were subsequently excluded for the following reasons: they were not focused on male involvement or related interventions; they were research protocols; they were qualitative papers; the aim of the study was not related to male involvement, attendance, accompanying and testing. 12 full-text papers were finally included in the review (Fig. 1).

### Study characteristics

All studies aimed to assess interventions to increase involvement of males in healthcare services offered in Antenatal Clinic (ANC) and the population included pregnant women who were both HIV positive and negative accessing healthcare services.

The included studies have been published from 2011 to 2018, and all the papers were related to SSA: Kenya (4), Malawi (3), Mozambique (1), Uganda (1), Nigeria (1), Tanzania (1) and South Africa (1). HIV/AIDS-related journals published most of the studies as Journal of Acquired Immune Deficiency Syndromes, AIDS and Behaviour, Journal of the International AIDS Society, Lancet HIV and Antiretroviral Therapy.

All studies focused on the male involvement issue, but there was no conventional way to evaluate and measure it [9]. In some studies, a male partner is involved if he escorts his partner to the antenatal clinic at least once [22, 23] or during a follow-up period of 4 weeks [24]. Nearly half of these studies did not explicitly define the concept. The adopted outcomes varied among the studies, but the recurrent outcomes were the antenatal attendance rate of the male partner (Proportion of pregnant women who attended ANC with their partners) and the male partner’s HIV test (Proportion of men who accepted routine antenatal HIV testing).

We included six experimental studies and six observational studies. These clinical studies were classified using an algorithm proposed by Grimes DA [25]. Among the experimental studies, six were randomized controlled trials. Among the observational studies, two showed a pre-post invention design [22, 26], two papers adopted a prospective cohort design [6, 27] and one a three different period interventions [28].

We described the papers (Table 1) based on the type of intervention: single intervention (7) and multiple integrated component (5) interventions.

**Table 1:** Articles included in the systematic review

Author, year	Country	Aim	Intervention type	Intervention period	Study design	Population	Main Outcomes	Main Results	Conclusions
*Akama E,* 2018	Kenya	To evaluate the impact of a male-centred Rapid Results Initiative (RRI)* on the PMTCT cascade focusing on: -HIV testing and linkage to care for patients HIV+;-keeping mothers alive through Antiretroviral Therapy Initiation and skilled deliveries; −uptake of Early Infant Diagnosis	Package of interventions employing an RRI approach including a male invitation letter, mentor mothers and peer educators, male-friendly services, fast-tracked at the clinic	-Baseline (January to March 2013) -During the RRI (April–June 2013) -Post-RRI (July–September 2013)	Pre- and post-intervention study	Pregnant women, partner, children in 116 antenatal clinics	1. Proportion of male partners accompanying their female partners to antenatal care (MI). 2. Proportion of partners receiving HIV testing. 3. HIV prevalence for pregnant women at ANC. 3. Proportion of women receiving skilled delivery services. 4. Proportion of HIV positive women receiving skilled delivery services. 5. Proportion of HIV positive women successfully linked to care. 6. Time to linkage to care. 7. Proportion of HIV exposed infants getting HIV testing	MI in ANC: from 7.4 to 54.2%(RD = 0.47, 95% CI 0.45–0.48) and 43.4% (RD = 0.36, 95% CI 0.35–0.37) The percent of male partners tested for HIV: from 5.4 to 50.1% (RD:0.45, 95% CI 0.43–0.46) and 38.6% (RD:0.33, 95% CI 0.32–0.34)	Male-centred RRI approach significantly contributed to increased uptake of HIV testing among male partners, earlier linkage to ART initiation among pregnant HIV-infected women, an increased proportion of women delivering in a health facility and infants. Strategies that deliberately address men’s own health needs appear promising at engaging men in PMTCT
*Aluisio AR,* 2016	Kenya	To assess the impact of partner ANC engagement on infant health outcomes of children born from HIV+ women	Invitation letters delivery	2009–2013	Multicentre prospective study	830 HIV positive pregnant women in 6 ANC	1.Male ANC Attendance 2.Male HIV test results per female report. 3.Infant Health Outcomes (HIV infection, infant mortality, HIV free survival)	N. of HIV-infected pregnant women: 830 (of which 11.2% reported no male partner, 25.9% refused partners participation) N. of male attended the ANC: 136 (26.2%) Number of men failed to attend: 383 (73.8%), of which 63 (16.5%) were surveyed through female partners. Previous male HIV test as per female report: Male attended ANC: 56.4% HIV + Male did NOT attend ANC: 56.4% HIV+ N. of infants born from women with ANC engagement: 132 (26.5%) Born without ANC engagement: 367 (73.5%)	Male ANC attendance was associated with improved infant HIV-free survival through six weeks of life. Infants lacking male ANC engagement had an approximately 4-fold higher risk of death or infection compared to those born to women with partner attendance (HR = 3.95, 95% CI:1.21–12.89, *p* = 0.023) (aHR = 3.79, 95% CI:1.15–12.42, *p* = 0.028)
*Aluisio AR,* 2011	Kenya	To investigate the relationship between male involvement in PMTCT services and infant HIV acquisition and mortality	Word of encouragement	1999–2002 Follow up until 2005	Prospective cohort study	456 HIV positive pregnant women in ANC	1. Number of male partners involved. 2. Correlates of male partner attendance. 3. Infant Outcomes (Mother to child HIV transmission; mortality; HIV-free survival)	Female with partner attendance: 31% Female without partner attendance: 54% Test HIV male partners: 56% HIV+. Association between male attendance and relationship status: women whose partners attended clinic were more likely to be in a monogamous marriage	Including men in antenatal PMTCT services with HIV testing may improve infant health outcomes. We observed a 63% less mortality risk among HIV-uninfected infants born to women whose partners attended clinic compared to those born to women whose partners did not attend
*Audet CM, 2016*	Mozambique	To evaluate the impact of a community-based intervention on male engagement in Antenatal Care.	The intervention has four components: 1. Involving of all TBAs** in MI 2. Developing Male Champions 3.“Male-friendly” clinical environment. 4. Couples Counseling Sessions	1 June 2012- 30 March 2014	Pre- and post-intervention study	5971 pregnant women (HIV status not considered) in ANC in four rural communities. Pre:1616Post: 5971	1. Uptake of provider-initiated counselling and testing among pregnant woman. 2. Male engagement in ANC (present at first visit, always present). 3. Uptake of ART.	The intervention was associated with increases in (post-intervention): (1) male engagement in ANC (5% vs. 34%; p < 0.001); (2) uptake of ART (8% vs. 19%; *p* < 0.001) (2) uptake of provider-initiated counselling and testing among pregnant woman (81% vs 92%; p < 0.001)	The study highlights the impact of increased male partner engagement on maternal testing. Therefore the adherence of women is higher with supportive male partners
*Byamugisha R, 2011*	Uganda	To evaluate the effectof a written invitation letter delivered to the spouses of women attending their first antenatal visit on couple attendance	*Intervention group*: invitation letter *Control group*: information letter or leaflet	October 2009–February 2010	A randomized, parallel-group, health facility-based intervention trial	1060 pregnant women enrolled: 530 for each group *Women attending the subsequent antenatal visits* Intervention group: 290 Control group: 310	Primary outcome: proportion of pregnant women who attended ANC with their partners during a follow-up period of four weeks. Secondary outcome: proportion of men who accepted routine antenatal HIV testing.	*Intervention group*: Couple antenatal attendance: 16.2% (intention to treat analysis). Partner accepted HIV test: 95.3% *Control group*: Couple antenatal attendance: 14.2% (intention to treat analysis). Partner accepted HIV test: 90.7%	The effect of the intervention and the control on couple antenatal attendance was similar in both arms. The majority (more than 90%) of the male partners who attended ANC accepted HIV counselling and testing
*Ezeanolue E, 2017*	Nigeria	To assess the effect of intervention Healthy Beginning Initiative (HBI) on the rate of HIV testing among male partners of pregnant women during pregnancy	Health Beginning Initiative is a congregation-based intervention. *Intervention group*: (1) community encouragement to participate in antenatal care, (2) education about the antenatal risk of HIV, (3) integrated testing to reduce stigma, (4) testing at convenient locations and (5) free testing. *Control group:* patients were referred for HIV testing at the nearest healthcare facility	Enrollment period: January 2013–September 2013. Follow-up of enrolled participants ended in August 2014.	Cluster randomized controlled trial	3047 pregnant women of which 2809 were married or partnered. Partners enrolled in 40 churches were 2498: − 1297 *Intervention group* − 1201 *Control group*	Completed HIV testing for male partners of pregnant women	Verified HIV testing rate among male partners was significantly higher in IG (84.0%) compared to CG (37.7%)	The study showed that the i*ntervention arm was more effective* than standard of care: male partners of pregnant women enrolled in the Intervention Group were 12 times more likely to have tested respect control Group.
*Jefferys LF, 2015*	Tanzania	To assess the acceptability and effectiveness of written invitations for male partners to attend Antenatal Care	Written invitation letter	March 2013–June 2013.	Prospective, longitudinal cohort	318 pregnant women	1. Association of socio-demographic indices with partner attendance rate. 2. Acceptability and effectiveness of invitation letters	−29% of women reported that they did not know their HIV status. -98% of women have delivered the letter to their partner. *Partner attendance rate:* 53% *Of these, 81% proceeded to counseling.* Of 170 male attending ANC: 79% did so after the first visit and 21% after the second invitation	This study demonstrated that written invitations for male partners to attend joint ANC and CVCT were well accepted by women attending ANC in Mbeya Region, Tanzania with significant differences in male attendance between our urban and rural settings. More than half of the women returned with their partner at a subsequent ANC visit
*Krakowiak D, 2016*	Kenya	To compare the effectiveness of **scheduled home visits** with pregnant women and their partners to written invitations encouraging men to return to the clinic for the next antenatal visit CVTC	*Intervention group:* invitation letter encouraging the male partner to attend the clinic at six months postpartum *Control group:* Scheduled home-based partner education and HIV testing visit	September 2013–June 2014	Randomized clinical trial	601 pregnant women *Control group:* 306 patients *Intervention group:* 295 patients	**HIV testing outcomes (%)**-Male partners HIV tested -Women knowing male partner status -No. of couples attending couple testing and counselling -Sero-discordant couples identified **Maternal and Child Health Outcomes**-Facility deliveries -Exclusive breastfeeding at 6-weeks postpartum -Family planning uptake	Male partners were more than twice [RR = 2.10; 95% CI: 1.81–2.42] as likely to have been HIV tested in the CG versus IG (87% versus 39%). Couples tested: CG arm was three times (RR = 3.17; 95% CI: 2.53–3.98) as likely to have been tested as a couple as the IG arm (77% versus 24%)	Home-based partner education and testing resulted in a more than 2-fold increase in male partner testing and HIV status disclosure and a higher than 3-fold increase in couple HIV testing and identification of HIV discordant couples when compared with partner invitation to attend antenatal care. The intervention did not result in higher uptake of maternal child health outcomes.
*Mphonda SM, 2014*	Malawi	To increase the number of couples attending an antenatal clinic and accessing PMTCT services	*Intervention:* **Period 1** (January 2007–June 2008): health talks performed by peer educators on the importance of involving men. **Period 2** (July 2008–September 2009): drama was introduced into patient waiting areas to deliver the same message. **Period 3** (October 2009–December 2009): male-friendly maternity wing.	January 2007–June 2008; July 2008–September 2009; October 2009–December 2009	Not defined (three interventions one after the other in the same hospital)	30,066 pregnant women: -period 1: 14,585 -period 2: 12,700 -period 3: 2781	-Percentage of pregnant females presenting as a couple-Male partners HIV status-Couples HIV status	-period 1: 0.7% (96) presented with male partner -period 2: 5.7% (732) presented with male partner -period 3: 10.7% (300) presented with male partner. The proportion of women attending ANC with a male partner increased from 0.7 to 5.7% to 10.7% over the three periods	Uptake remained sub-optimal, and are needed strategies community-based as a complementary to any facility-based component. Authors recommended the expansion of peer counselling programs with drama and structural changes in all facilities in Malawi
*Nyondo AL, 2015*	Malawi	To evaluate the efficacy and feasibility of an **invitation card** to the male partners as a strategy for male involvement in PMTCT services	*Intervention group:* invitation letter *Control group:* women use “word of mouth” to involve male partners. The message was similar	Recruitment period: 14 June- 17 December 2013. Follow up activities completed on 24 February 2014	Randomized open-label controlled trial	462 pregnant women attending antenatal care without a male partner *IG:*230 *CG:* 232	*Primary outcome:* proportion of pregnant women who reported to the study clinic with their partners, for PMTCT services following use of an invitation card after two visits.	Of the 462 women: 109/462 (23.59%) came back with their partners at one visit. Concerning each study group reported with their partners: IG: 65/230 (28.26%) CG: 44/232 (18.97%)	Invitation card increases the proportion of women who are accompanied by their male partners for the PMTCT
*Rosenberg NE, 2015*	Malawi	To compare two strategies for recruiting male partners for Couple HIV Testing and Counseling: invitation-only versus invitation plus tracing	*Intervention group:* Invitation card plus partner’s tracing if he did not present within one week. A community health worker can make three phone attempts, followed by three community attempts. *Control group:* Invitation card	March 2014–October 2014	Unblended, randomised, controlled trial	200 HIV-positive pregnant women were enrolled and randomly assigned to IG: 100 CG: 100	*Primary outcomes:* Proportion of couples who presented to the clinic together and received CHTC^§^.*Secondary outcomes*: -HIV status of men-Proportion of HIV-positive men newly diagnosed -Proportion of newly diagnosed HIV-positive men linked to care	*Intervention group:* 74% (74/100) couples presented for CHTC 46% (15/33) new HIV+ men linked to care *Control group:* 52% (52/100) couples presented for CHTC 19% (5/26) new HIV+ men linked to care	Invitation plus tracing results highly effective at increasing CHTC uptake
*Weiss SM, 2014*	South Africa	To determine whether men’s participation in the intervention would significantly impact PMTCT uptake compared to male attendance at antenatal visits only	*Intervention group:* Partner Plus intervention consisted of a couples’ behavioural HIV risk reduction intervention: 4 weekly session utilizing a cognitive-behavioural skill training approach	Recruitment: January 2010–July 2011	Randomized control trial (pilot study)	478 participants (238 pregnant women and 239 men)	-Baseline and post-intervention data about HIV serostatus. -Male Attendance -HIV and PMTCT knowledge.-PMTCT-specific knowledge	Male attendance (involvement) increased in both conditions, but no significant difference between the intervention and control conditions were found (t _201.5_ = 1.76, p = .08). No difference in the number of visits attended by men (t _223_ = 1.3, *p* = .18)	Male attendance at antenatal visits was high in both experimental and control conditions, but only the experimental group demonstrated significant improvements in PMTCT outcomes, confirming the need for psychoeducational programs to actively engage both HIV-seropositive pregnant women and their male partners in the PMTCT process.

*RRI is a management concept combining best practices from organizational psychology, change management and capacity building, **TBA: Traditional Birth Attendants; ^§^ Couple voluntary counselling and HIV testing; Intervention group (IG); Control group (CG)

### Single intervention

We considered researches that evaluated only one strategy to improve the participation of men as a single intervention study. Seven studies met this criteria, four of these were randomized controlled trials. Six interventions adopted the use of an invitation letter and one intervention introduced word of encouragement to involve partners.

### Interventions based on invitation letter

Six studies adopted a strategy based on invitation cardin Malawi (2), Kenya (2), Tanzania (1) and Uganda (1). The interventions varied from 3 months to 4 years and were provided through antenatal care visits. Four out of six studies were randomized controlled trials.

In all six studies men were encouraged to attend the antenatal care through invitation card or letter sent via their partners. We considered the invitation card or letter as the standard of care of each study described in this session.

Two studies evaluated the use of an invitation letter: in Aluisio et al. [29], among 830 enrolled women, 62.5% (519/830) agreed to involve males and 26.2% (136/519) of partners attended ANC, while in the paper of Jefferys et al. [27] partner attendance rates reached 53.5% (170/318) during the study period.

Four RCTs compared the invitation card to word of mouth [30], information letter [24], home visits [31] and invitation card plus partner tracing [32]. Word of mouth seems less effective than SC. Among 462 randomized women, 28.26% (65/230) of the women came back with their partners in the standard of care group compared to 18.97% (44/232) in the word of mouth group. Therefore, women in the SC group were more likely to be accompanied by their male partners (intention to treat analysis RR: 1.49 (95% CI:1.06–2.09); *p* = 0.02; per protocol analysis RR: 1.43, (1.03–1.97); *p* = 0.03) than women in the word of mouth group [30].

In the study performed by Byamugisha et al. [24], the use of the invitation letter is compared to a leaflet related to the healthcare services delivered in the antenatal clinic. The researchers found out similar effect in both group: 16.2% (86/530) of pregnant women attended ANC with their partners in the standard of care and 14.2% (75/530) in the leaflet group (unadjusted OR = 1.2; 95% CI:0.8–1.6; intention to treat analysis).

Krakowiak et al. [31] enrolled 1101 pregnant women attending their first antenatal visit at Kisumu Country Hospital (Kenya). Women were randomly assigned to two groups: in first group, they received a scheduled home-based partner education including HIV testing (within 2 weeks of enrolment); in the second group, the women received an invitation card asking the asking partner to attend the clinic for HIV couples counselling and testing visit (6 months post-partum). Male were more than twice [RR = 2.10; 95% CI: 1.81 to 2.42] as likely to have been HIV tested in the scheduled home visits [87% (233/247)] as compared to standard of care [39% (108/240)] at 6 months post-partum. In this research study, couples testing was proposed to couples. In the home visit group, couples were three times (RR = 3.17; 95% CI: 2.53–3.98) as likely to have been tested than the standard of care group (77% versus 24%, respectively).

The RTC conducted by Rosenberg et al. [32] compared the invitation plus tracing intervention (first arm) to the standard of care (second arm). The women received an invitation card and in the first arm the partners were traced if they did not come to the centre for couples HIV testing and counselling (CHTC). A community health worker outlined the male partners through phone calls and community encounters. In the standard of care group, only 52% (52/100) of couples presented for CHTC, while in the invitation plus tracing, 74% presented for CHTC (74/100).

### Word of encouragement intervention

In the study of Aluisio et al. [6], women were encouraged to invite their partners for participation. The article examined the interrelation between male involvement and infant HIV acquisition and mortality through a prospective cohort study. The researchers used a strategy to raise partner awareness in women. Men were proposed Voluntary Counselling and Testing (VCT) and those who accepted were invited to come back after 1 week for the test’s results and counselling. Among the enrolled HIV-infected pregnant women (456), 31% (140/456) of partners attended the antenatal clinic, 54% (75/140) were tested, and 56% (42/75) were HIV positive.

### Multiple integrated components

Five papers included multiple integrated components. These interventions showed an effect on multiple levels of the health behaviour change model for HIV prevention and AIDS care. This socio-ecological model [33] presents all the factors influencing HIV-related behaviour and considers the complex interaction between individual, relationship, community, and societal factors [34]. In the review process, we considered this model.

As described in Fig. 2, all studies focused on individual interventions and the institutional/health system level, only two focused on the community level, and two focused on the interpersonal/network level.

The studies were classified by dividing interventions between those who target men and offer health education [28, 35, 36] and those who aim to train professional figures in the healthcare sector working with the MI target [26]. Akama et al. [22] described more complex multi-faceted interventions.

Among these five studies, two were RCT [35, 36], two were pre-post intervention design [22, 26] and one consisted of three interventions, one after the other in the same hospital [28].

In the pre-post intervention study, Audet et al. [26] designed four component interventions including involvement of Traditional Birth Assistance in MI, developing male champions figures, creating male friendly clinics and implementing couple sessions. Five thousand nine hundred seventy-one pregnant women were enrolled; during the period, rate of male accompaniment at the first or at any ANC appointment increased (respectively 5% vs. 34% *p* < 0.001 and 10% vs 37% p < 0.001), and an increment in the rate of HIV testing for male partners during ANC was observed (9% vs 34%; p < 0.001). Women who were accompanied by partners to the ANC received more likely HIV testing (aOR: 5.98; 95% CI: 4.50–7.94; p < 0.001), attended three ANC visits (aOR: 1.26; 95% CI: 1.10–1.45; p < 0.001), and delivered in a health facility (aOR: 1.26; 95% CI: 1.08–1.47; *p* = 0.003).

In the RCT conducted by Ezeanolue and colleagues [35], a sample of pregnant women attending church activities was analysed. The effectiveness of a congregation-based Healthy Beginning Initiative versus a clinic-based approach on the rates of HIV testing was investigated. The intervention provided men with 1) community support to participate in antenatal care; 2) education about HIV infection and antenatal risk; 3) free laboratory exams during a church-organized ‘baby shower.’ The intervention was intended to promote HIV testing of male partners, enhance tracking and strengthen linkage to the health service.

In this study,40 churches in Nigeria were randomized, 3047 women and 2498 partners were enrolled during prayer sessions. The intervention arm was more effective than standard of care, in fact 84.0% (1089/1297) of males undertook HIV testing versus 37.7% (453/1201) (χ^2^ = 564.48, *p* < .001) in the standard of care. Male partners who received the interventions underwent HIV testing12 times more often compared to the standard of care (aOR = 11.9; *p* < .01).

Mphonda et al. [28] introduced three interventions: one after the other in the same hospital from January 2007 to June 2008, from July 2008 to September 2009 and from October 2009 to December 2009. The interventions included health talks, drama in the patient‘s waiting-areas and an only-for-men clinic area. An increment from 0.7% (96/14585) to 5.7% (732/12700) to 10.7% (300/2781) was registered in the number of men accompanying their spouse to ANC over the three periods.

The RCT of Weiss et al. [36] compared an intervention to reduce behavioural HIV risk in couples combined with a medication adherence intervention to standard of care. This intervention was based on a cognitive-behavioural approach through 4 weekly sessions. An increment in male attendance was observed under both conditions, an increment in knowledge of HIV and PMTCT topics was observed only in the group involved in the intervention. Looking at the number of visits attended by women, no significant difference was observed between the two groups: 5.7 ± 1.5 for the intervention group versus versus 2.2 ± 1.8 (t _201.5_ = 1.76, *p* = .08) for control group. The same in the male attendance to visits (t _223_ = 1.3, *p* = .18). Not even differences were noted according to provenience (rural vs non-rural) and occupational status (employed vs unemployed).

Akama et al. [22] described a package of interventions, that showed effect at community, health facility and individual levels: community mobilization, health education in the waiting areas, invitation letters, peer educators and mentor mothers equipped with mobile phones, text reminders and phone calls to male partners, leveraged existing community units for mobilization, fast tracked clinic visits and abbreviated medical check-ups.

The study involved 116 ANC in Western Kenya, assessing male involvement pre and post- intervention. An increment from 7.4% to 54.2% (RD = 0.47, CI 0.45–0.48) was observed in the number of men escorting their partners to the clinic and receiving counselling at baseline and during intervention respectively. A further reduction to 43.4% (RD = 0.36, CI 0.35–0.37) was observed in the post-intervention period.

In Table 2, we describe the outcomes used in all the studies included in the systematic review.

**Table 2:** The outcomes related to men involvement

	Proportion of pregnant women who attended ANC with their partners	Proportion of men who accepted routine antenatal HIV testing	HIV status of men/newly diagnosed	Proportion of newly diagnosed HIV-positive men linked to care	HIV testing outcomes*	Maternal and Child Health Outcomes**
Single Intervention						
Interventions based on Invitation letter						
Aluisio et al. [29]	X					
Byamugisha et al. [24]	X	X				
Krakowiak et al. [31]			X		X	X
Jefferys et al. [27]	X					
Nyondo et al. [30]	X					
Rosenberg et al. [32]	X		X	X		
Intervention based on Word of encouragement						
Aluisio et al. [6]		X	X	X		X
Multiple integrated components						
Akama et al. [22]	X	X			X	X
Audet et al. [26]	X				X	
Ezeanolue et al. [35]		X				
Mphonda et al. [28]	X				X	
Weiss et al. [36]	X				X	

* Male HIV tested, Female knows male status, tested as couple Serodiscordant couples identified

** In Krakowian et al. [31] “Facility delivery, Exclusive breastfeeding at 6-weeks postpartum, Exclusive breastfeeding at 6-months postpartum then family-planning hormonal, intrauterine device, or sterilization use at six weeks postpartum and 6 months postpartum”. In Aluisio et al. [6] and Akama et al. [22] child health outcomes refer to mortality and HIV free survival and EID uptake infants (0–8 months), respectively

## Discussion

In the present study, we conducted a systematic review of all the interventions aimed to increase the involvement of male partners in antenatal clinics. The characteristics of the interventions did not allow us to perform a meta-analysis, so we narratively described the interventions. We were interested in the effectiveness of male involvement interventions in term of attendance (as the proportion of men escorting their female pregnant partners to visits to ANC visits) and, where not present, male HIV testing. We highlighted only these outcomes in the results, although other outcomes were evaluated in the included studies. During the review process, we screened 1694 studies, and we included 12 papers.

The studies were conducted in 7 different Sub-Saharan countries, and population characteristics were not comparable, but the interventions including comparator arms (randomized controlled trial) or pre-post intervention studies seemed effective, excluding the couple behavioural HIV risk reduction intervention where male attendance increased in both arms [36].

From the recent literature research, three systematic reviews emerged which presented different strategies to involve men in maternal and childcare. The systematic review performed by Tokhi et al. [37] aimed to measure the effect of interventions to increase male involvement during pregnancy and first year of life in order to improve maternal and newborn health. The included studies (13) showed that the engagement of men in maternal and new-born health positively impacted on relationship dynamics.

The review of Takah et al. [38] including 17 studies (4 randomised trials and 13 observational studies) based in SSA, aimed to identify the strategies to improve men involvement in PMTCT programs and the consequent effect on adherence to ART in positive pregnant women. Takah et al. included also studies not clearly designed to.

The meta-analysis showed that community interventions were more effective than invitation letter in engaging men and adherence in PMTCT. The invitation letter did not demonstrate an effect.

The systematic review performed by Hensen et al. [39], focused on the involvement of men in HTC (HIV Test and Counseling) services in SSA and included 15 papers. Hensen et al. identified several number of interventions effective in increasing HTC services uptake by men, even when general population was targeted.

Furthermore, community-based programmes showed to be effective in increasing HTC access at population level.

These reviews represent an essential state of the art, but our review is focused on studies referring only to interventions or strategies that explicitly promote male partners in PMTCT, even if the final outcomes of the interventions is women’s health.

Among the single interventions, the randomized controlled trial comparing the invitation card strategy to home visit was more effective for delivery of couples’ HIV testing at home than the others. This intervention seemed more promising than word of mouth, information letter and the invitation plus tracing strategy. The partners’ attendance rate was lower in SC than in the intervention arm: leaflet (14.2% vs 16.2%), home visit (39% vs 87%) and invitation card plus partner tracing (52% vs 74%). Home visit strategies seemed the most effective [31].

The multi-component intervention showed higher effectiveness in terms of male accompaniment at the first ANC than the single interventions. These studies would likely to produce a change mainly at the individual, Institutional/health system level, and slightly at the interpersonal/network, community and structural levels.

Although male involvement increased in some studies [22, 26, 35], Akama et al. [22] and Audet et al. [26] assessed effective interventions in order to increase the number of men escorting their female partners at ANC visit (pre-intervention 7.4%- post-intervention 43.4%; pre-intervention 5%- post-intervention 34%, respectively) and Ezeanolue et al. [35] evaluated the percentage of men tested for HIV (intervention 84% vs standard of care 38%) .

As shown in Fig. 2, Akama et al. and Ezeanolue et al. [22, 35] studies tried to assess the impact of the interventions in multiple levels of the Socio-ecological model [33] compared to other studies. The modern health promotion theory suggests multifaceted intervention target obstacles and facilitators are comparatively more effective [32].

Regarding outcomes assessed in the studies, many of the interventions evaluated the number of male partners escorting their female partners to ANC visit and accepting to be tested for HIV.

The number of studies identifying MI as a necessity was very high, while the number of studies able to identify the most effective interventions or strategies was somewhat limited. From this, we deduce the need to carry out more studies in this area, and to define in a more precise way standard indicators to measure MI.

### Limitations of the study

The search was comprehensive because the primary databases were reviewed. Two reviewers conducted independent search and screening of studies to reduce bias. Our systematic review has several limitations. Due to the resources available, we did not include a search for papers published in the grey literature. Moreover, we did undertake a search in languages other than English, and we did not include papers written in other languages as French, Spanish, Portuguese and Dutch.

Therefore, during our research, we realized that some studies are underway because study protocols have been published, but there are not yet published results (Trial registration number: ISRCTN18421340 and NCT02085356).

There are also limitations relating to our selection criteria. We decided to include only papers where the research was to improve male partner involvement, although we found studies where the objective was not to evaluate male involvement, but MI was reported together with other indicators.

The included studies were heterogeneous since they showed variability in the studied population, interventions, location and social context (country, urban or rural area), measured outcomes and study design.

## Conclusion

A low number of studies aimed to improve male involvement was found in our search. The need to define clearer indicators for measuring male involvement is very apparent. There is also the need for development of more studies that have lower heterogeneity in regard to design, outcomes and geographic location. The review highlights the importance of male involvement in HIV cascade for pregnant women in countries with a significant HIV incidence. HIV counseling and testing is fundamental in the care process but is also very important for the continuum of the care after pregnancy both for women and their partners. The efficacy and effectiveness of implemented interventions need to be assessed over the long term.

## Supplementary information

**Additional file 1: Table S1.** Search terms. List of the electronic search strategies. **Table S2:** PRISMA 2009 Checklist PRISMA checklist items.

## Abbreviations

ANC: Antenatal Clinic
aOR: Adjusted Odd Ratio
ART: Antiretroviral therapy
CG: Control group
CHTC: Couples HIV testing and counselling
CI: Confidence Interval
HTC: HIV Test & Counseling
IG: Intervention group
MI: Male involvement
MTCT: Mother-to-Child Transmission
OR: Odd Ratio
PMTC: Prevention of Mother-to-Child Transmission
pPTCT: Prevention of Parent to Child Transmission
PRISMA: Preferred Reporting Items for Systematic Reviews and Meta-Analyses
RCTs: Randomized Controlled Trials
RD: Risk Difference
RR: Risk Relative
SC: Standard of Care
SSA: Sub-Saharan Africa
VCT: Voluntary Counselling and Testing
